# What serial homologs can tell us about the origin of insect wings

**DOI:** 10.12688/f1000research.10285.1

**Published:** 2017-03-14

**Authors:** Yoshinori Tomoyasu, Takahiro Ohde, Courtney Clark-Hachtel

**Affiliations:** 1 Department of Biology, Miami University, Pearson Hall, 700E High Street, Oxford, OH 45056, USA; 2 Division of Evolutionary Developmental Biology, National Institute for Basic Biology, 38 Nishigonaka Myodaiji, Okazaki 444-8585, Japan; 3 Department of Basic Biology, School of Life Science, SOKENDAI (The Graduate University for Advanced Studies), 38 Nishigonaka Myodaiji, Okazaki 444-8585, Japan

**Keywords:** insect wing origin, evolutionary novelty, serial homology, evo-devo, vestigial, Hox

## Abstract

Although the insect wing is a textbook example of morphological novelty, the origin of insect wings remains a mystery and is regarded as a chief conundrum in biology. Centuries of debates have culminated into two prominent hypotheses: the tergal origin hypothesis and the pleural origin hypothesis. However, between these two hypotheses, there is little consensus in regard to the origin tissue of the wing as well as the evolutionary route from the origin tissue to the functional flight device. Recent evolutionary developmental (evo-devo) studies have shed new light on the origin of insect wings. A key concept in these studies is “serial homology”. In this review, we discuss how the wing serial homologs identified in recent evo-devo studies have provided a new angle through which this century-old conundrum can be explored. We also review what we have learned so far from wing serial homologs and discuss what we can do to go beyond simply identifying wing serial homologs and delve further into the developmental and genetic mechanisms that have facilitated the evolution of insect wings.

## Introduction

The acquisition of wings is considered a major driving force for the success of insects, yet the evolutionary origin of this important novel structure remains one of the biggest conundrums in biology. Over a century of investigations into this question have resulted in two prominent hypotheses on the evolutionary origin of insect wings: the tergal hypothesis and the pleural hypothesis. The tergal origin hypothesis (also known as the paranotal lobe hypothesis) proposes that wings originated from expansions of the dorsal body wall (terga), whereas the pleural origin hypothesis essentially proposes that wings evolved from pleural (lateral body wall) tissues and their associated branches (exites) (see 1 and 2 for a review of these two hypotheses) ^1, 2^. Recently, studies using molecular and evolutionary developmental (evo-devo) analyses have provided a new view through which this conundrum can be assessed. A summary of the history of the wing origin debate and an overall perspective on how the application of evo-devo approaches to this question can lead to new insights on the evolutionary origin of insect wings have previously been reviewed ^3^. Here, we focus our discussion on how the identification of wing serial homologs through developmental approaches will help us to explore the history and origin of insect wings and on what we have learned so far. Then we discuss the challenges we face with evo-devo approaches and how we can overcome these challenges.

## What is serial homology and why is it helpful to understand the origin of insect wings?

The definition of serial homology (and homology in general) in evolutionary biology has been quite controversial (see 4 for a comprehensive review of the homology concept) ^4^. Fortunately, the situation is much less complicated in insects because of their metameric body plan. The ancestral arthropod body likely consisted of repeats of fairly uniform segments, each of which possessed a common set of structures (such as a pair of legs) ^5^. In this body plan, these structures on different segments are considered to be serially homologous to each other, as the development of these structures was likely orchestrated by the same developmental system. Throughout arthropod evolution, these serially homologous structures have often undergone segment-specific modification or suppression, resulting in serially homologous structures with different morphologies and different functions (for example, legs and antennae) ^5^ (also, see Box 3 of 3). Why is the identification of wing serial homologs useful to explore the origin of insect wings? The key concept is that serial homologs have undergone differing degrees of evolutionary modification in different segments (that is, some serial homologs may retain more ancestral morphologies than others). Therefore, by identifying various structures that are serially homologous to wings and comparing their development with that of wings, we may be able to reconstruct a transition series from the origin tissue to the wing and therefore identify the key developmental events that led to the acquisition of wings.

The concept of gaining insights into evolutionary transitional states of wings through wing serial homologs is not new. In fact, this has been the main approach (besides paleontological approaches) used by insect wing origin studies ^3^. However, identification of wing serial homologs has been limited, mainly because the search has been for structures morphologically similar to wings in non-winged segments (and also because of the subjective nature of deciding which structures are “similar” to wings). Since there are no obvious “wing-like” structures outside of the second and third thoracic segments (T2 and T3) in insects, only a handful of structures (such as mayfly gills and termite paranotal expansions) have been proposed to be wing serial homologs ^6– 10^. But, as mentioned above, serially homologous structures can lose morphological similarity, even though they have historical common ancestry (namely, they can be deeply or cryptically homologous ^11^). Therefore, it is possible that wings are so drastically diverged from other serially homologous structures that we are not able to recognize them as wing serial homologs. The application of molecular and functional approaches (an evo-devo approach) may allow us to identify “hidden” wing serial homologs in wingless segments, which could provide us with critical information to unveil the origin of insect wings.

**Figure 1.**
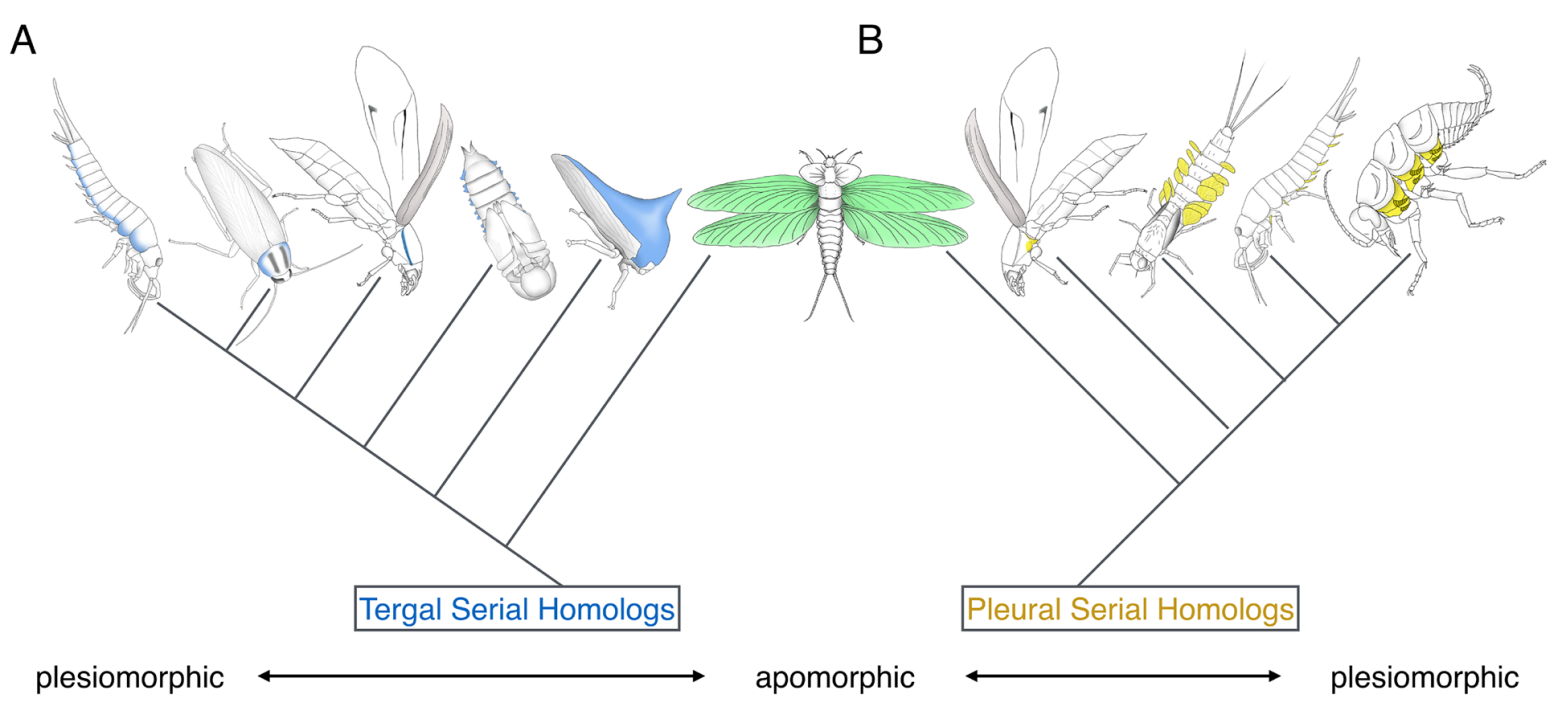
Evolutionary relationship among the identified wing serial homologs. ( **A**) Dorsal/tergal wing serial homologs. ( **B**) Lateral/pleural wing serial homologs. The wing serial homologs included in the figure are ( **A**) bristletail tergal edge, cockroach lateral pronotum, beetle T1 carinated margin, beetle pupal gin-trap, and treehopper helmet and ( **B**) two beetle T1 pleural plates, mayfly nymphal gill, and bristletail stylus. Pleural plates with exites of a hypothetical hexapod ancestor are also included in ( **B**) (although the presence of pleural exites in some fossil insects is currently under debate in the paleontology community ^60– 62^).

It is important to emphasize that we are not suggesting that the non-winged segments historically possessed wings. Our understanding is that there has been no fossil evidence supporting the presence of *bona fide* wings in segments other than the two thoracic segments in the history of hexapod evolution (Palaeodictyoptera had wing-like structures in T1, but these winglets are not usually considered true wings ^12^). Instead, we are suggesting that there are tissues in these wingless segments that have common ancestry with insect wings and that wings are “apomorphic” (that is, evolutionarily diversified from the ancestral state) among serially homologous tissues ( Figure 1).

## An evo-devo-based strategy to identify serially homologous structures

There are two evo-devo concepts that are crucial for the identification of serially homologous structures in insects: marker genes and Hox region selector genes. **Marker genes** in evo-devo studies are the genes that are expressed uniquely in a set of related tissues and thus are often useful to investigate the evolutionary and developmental relationships among tissues. Marker genes can be divided into two different classes. The first class is composed of the genes that are directly involved in the function of terminally differentiated tissues, such as pigmentation and cuticle genes for epidermis and the opsin genes for the eye ^11, 13^. Marker genes belonging to this class are also known as effector or realizator genes, which often code for enzymes and structural proteins ^14^. Since their expression signifies unique cellular and tissue functions, marker genes belonging to this class are useful to determine the type of terminally differentiated cells and tissues. The second class consists of the genes that orchestrate the entire development of a certain tissue. The expression of this class of marker genes tends to start at the beginning of the tissue induction process and often continues throughout the development of the resulting tissue. Genes belonging to this class are also called master genes, identity selector genes, or character identity genes ^4, 15^. Many of the genes in this class encode transcription factors.

As discussed in the previous section, tissue function can diverge among serially homologous structures, which means that the expression of the first class of marker genes likely differs even among serially homologous tissues if the function of these tissues is different. On the other hand, the same “master” gene (the second class of marker genes) often orchestrates the development of tissues that are serially homologous to each other; thus, this type of marker gene is useful for investigating the history of the tissue and for identifying serial homologs that are morphologically diverged. The wing and leg marker genes discussed in this review are in the second class of marker genes.

**Hox genes** are the region selectors responsible for the individualization of the otherwise uniform segments and segmentally repetitive structures (that is, serial homologs) ^5, 16, 17^. Hox genes achieve this task via at least three distinct modes of action: (i) modification of pre-existing structures, (ii) suppression of pre-existing structures, and (iii) induction of *de novo* structures (also, see Box 3 of 3). In the first mode (Hox action 1), the marker gene expression is often kept among the modified serially homologous structures. In contrast, in the second mode (Hox action 2), the tissues that express the marker gene itself are missing. The third mode (Hox action 3) is rather unique since *de novo* structures induced by this Hox action usually lack serial homologs in other segments. It is important to note that Hox genes are usually not involved in the induction of structures that are present in multiple segments as serial homologs, but instead modify the structures induced by the developmental system that is shared among the serial homologs. Therefore, removing Hox function (that is, loss-of-function of Hox) will often allow us to strip away the modifications applied to the serially homologous structures (via Hox action 1), revealing serial homologs that are otherwise morphologically distinct and difficult to recognize.

## Serial homology of insect ventral appendages

Let us first use the legs and their derivatives (ventral appendages) in *Drosophila* as an example to discuss how we can apply the above-mentioned evo-devo concept to the identification of serially homologous structures. Head and thoracic segments of *Drosophila* possess a pair of appendages on their ventral side. In the head segments, these appendages form a series of mouthparts as well as antennae. On the other hand, each of the three thoracic segments possesses a pair of legs, with minor modifications making the three pairs of legs slightly different from each other. *Distalless* ( *Dll*), which codes for a homeodomain transcription factor, is one of the important marker genes for leg development ^18^. *Dll* is expressed in mouthparts, antennae, and legs ^18^, and these ventral appendages can transform into one another upon various Hox mutations (including both gain-of-function and loss-of-function (LOF) mutations) ^5^. These outcomes indicate that the head appendages and legs are serially homologous and that Hox genes modify these serially homologous structures in a segment-specific manner (Hox action 1). In contrast, there is no ventral appendage in the abdominal segments of *Drosophila*, as Hox genes suppress the induction of ventral appendages ^5, 19^. This is also apparent from the fact that *Dll* is never induced in the abdominal segments ^18^. Upon Hox LOF mutations, a new set of *Dll*-positive cells are induced in the abdominal segments, producing ectopic leg primordia ^19^. Through this outcome, we can conclude that there are no leg serial homologs in the *Drosophila* abdominal segments (Hox action 2).

Although this configuration was initially recognized in *Drosophila*, later studies showed that it is conserved among other insect orders (for example, see an astonishing transformation of all ventral appendages into antennae, along with the ectopic induction of antennae throughout abdominal segments, in *Tribolium* ^20^). Interestingly, ventral appendages in the abdominal segments have been regained in some insect lineages (such as the prolegs of lepidopteran larvae). The tissues that give rise to these ventral appendages are accompanied by *Dll* expression ^21^, further supporting the idea that the presence of serial homologs corresponds to the expression of marker genes. In addition, studies in horned beetles provided an example of the third Hox action in regard to the ventral appendage. Beetle horn has *Dll* expression ^22^. However, this has been achieved via co-option ^22^, and removing the responsible Hox gene will simply remove the horn ^23^. Hence, we can conclude that horn formation has evolved via co-option of the leg gene network (including *Dll*) under the control of the Hox gene and thus that there are no horn serial homologs in other segments.

## The lack of wing serial homologs in dipteran insects

At least in *Drosophila*, the situation of wings and their derivatives (dorsal appendages) somewhat parallels the situation of the ventral appendages. As mentioned, morphologically, wings are unique to the two thoracic segments (T2 and T3) in extant insects, including *Drosophila*. Among the several wing genes identified from *Drosophila* studies, *vestigial* ( *vg*) is often considered one of the most critical wing marker genes because of its wing-specific function during the development of epidermal structures (although *vg* does have additional functions in tissues outside of wings and halteres, such as muscle ^24– 26^) and its ability to induce ectopic wings when overexpressed ^27– 30^. In *Drosophila*, dorsal appendage primordia (wing and haltere imaginal discs) are induced during embryogenesis. These imaginal discs in T2 and T3, along with a pair of residual cell clusters in T1, are the *vg*-positive epidermal tissues in *Drosophila* ^27, 31^. In contrast, the segments outside of the thorax do not have *vg*-positive epidermal tissues that contribute to adult morphology ^27^. Because *vg*-expressing imaginal tissues are missing in the non-winged segments, it has been considered that the induction of wing-related structures (that is, wing serial homologs) is suppressed in these segments, similar to the absence of the leg serial homologs in the abdominal segments of *Drosophila* (Hox action 2).

## The discovery of wing serial homologs outside of T2 and T3 in beetles and other insects

Because of this well-established *Drosophila* paradigm regarding the unique presence of wing-related tissues in T2 and T3, it came as a surprise when additional *vg*-dependent epidermal tissues were found in non-winged segments in beetles. Ohde *et al.* found that the formation of some of the dorsally originated tissues, such as hypomeron in T1 and gin-traps (pupal defensive structures) in the abdominal segments, is *vg*-dependent in the mealworm beetle, *Tenebrio molitor* ^32^. Clark-Hachtel *et al*. identified that there are two distinct *vg*-dependent tissues in T1 of the red flour beetle, *Tribolium castaneum*: (i) the carinated margin, a dorsally originated tissue that corresponds to the edge of the hypomeron, and (ii) two pleural plates (posterior trochantin and the epimeron), tissues that are considered to have originated from ancestral proximal leg segments (and therefore are pleural in nature) ^33^. Furthermore, in both studies, these *vg*-dependent tissues were shown to transform into wings upon Hox LOF mutation. These results strongly suggest that, although their shape and function are drastically different from those of wings, the *vg*-dependent tissues found in the non-winged segments of beetles are wing serial homologs.

Additional evidence for the presence of wing serial homologs in non-winged segments comes from the outcome of the abdominal Hox gene analysis in beetles. Knocking down the abdominal Hox genes during the larval stage in *Tribolium* results in the induction of almost complete wing tissues throughout the abdominal segments ^34^. However, legs were never ectopically induced in the same condition. This indicates that there are tissues which can transform into wings upon Hox LOF mutations (that is, wing serial homologs), while there are no leg serial homologs in the abdominal segments which can transform because those tissues are never induced (suppression via Hox action 2). Therefore, these results further support the idea that wing serial homologs are present in the non-winged segments and that the Hox action on the wing serial homologs is different from that on the leg serial homologs in the abdominal segments in beetles.

The presence of wing serial homologs appears to be widespread in various insect orders, including hemimetabolous orders. The impressively exaggerated treehopper helmet was shown to have expression of *nubbin* ( *nub*) ^35^, another critical wing marker gene ^36^. Although the *vg* dependency of this tissue has yet to be tested, given that the treehopper helmet is an expansion of the prothoracic tergum ^37, 38^, it is likely that the helmet is homologous to one of the two *vg*-dependent tissues identified in beetles and thus at least “partially” serially homologous to wings (also, see 3 and 33 for more discussion on the partial homology between treehopper helmets and wings). In addition, through genetics and genomics approaches, Medved *et al*. have provided functional evidence supporting the presence of wing serial homologs in the T1 of the milkweed bug, *Oncopeltus fasciatus* ^39^. More recently, Elias-Neto and Belles also reported the presence of two distinct wing serial homologs in the T1 of the German cockroach, *Blattella germanica* ^40^.

Why, then, were these wing serial homologs not discovered in *Drosophila*? There is no doubt that insect wing development has been studied most thoroughly in *Drosophila*, which has led to an excellent understanding of the molecular basis underlying wing development ^41, 42^. However, in regard to wing serial homologs, the unique dipteran body plan was the problem. Most of the dorsal T1 structures (and probably some lateral structures as well) are missing from adult *Drosophila* ^43^. Also, the entire adult abdomen is formed from a set of unique tissues, called histoblasts ^43^. Owing to this highly derived body plan, *Drosophila* appears to have lost most of the *vg*-dependent epidermal tissues that contribute to the adult morphology in the non-winged segments. This signifies the importance of investigating a wide variety of taxa to gain a comprehensive view of insect wing evolution. In summary, wing serial homologs appear to be widespread in various insect orders, and the evo-devo approach outlined above provides a new way of identifying them: namely, surveying for *vg*-positive epidermal tissues followed by Hox transformation studies to test whether the identified tissues transform into wings.

## What wing serial homologs have told us so far

The seminal studies in non-insect arthropods performed by Averof and colleagues provided evidence for a pleural origin of insect wings ^44, 45^. Later, through expression analyses in basal insects, Niwa *et al*. proposed a dual (pleural + tergal) origin of insect wings ^46^. Studies on wing serial homologs in winged species (discussed above) have also resulted in somewhat varying conclusions on the origin of insect wings. The identification of dorsal wing serial homologs in the T1 (hypomeron) and the abdomen (gin-traps) of *Tenebrio* beetles provided evo-devo support for a tergal origin of insect wings ^32^. Interestingly, two sets of wing serial homologs were identified in the T1 of *Tribolium*: one tergal and the other pleural in nature. The merger of these two wing serial homologs (both tergal and pleural) appears to be essential for the formation of ectopic wings upon Hox LOF mutation, suggesting a dual origin of insect wings ^33^. In more basal insects, Elias-Neto and Belles concluded that both tergal and pleural tissues contribute to the formation of an ectopic wing on T1 in *Blattella* ^40^, and Medved *et al*. provided transcriptional support for the contribution of both tergal and pleural tissues to the wings of *Oncopeltus* ^39^. These results may provide further support for a dual wing origin.

Two important messages can be obtained from the wing (serial) homolog studies: (i) as mentioned, wing serial homologs are widespread, and (ii) wing serial homologs can have drastically different morphologies from each other. For example, in regard to the wing serial homologs of tergal origin, they can be lateral expansions of dorsal body wall or an elaborated helmet in T1, gin-traps (modified body wall) in the abdomen, and wings in T2 and T3. Given that many of the dorsal wing serial homologs are modified body wall structures, the body wall character state appears to be more plesiomorphic (that is, retaining ancestral morphologies) among the dorsal wing serial homologs (albeit with varying degrees of modification), whereas insect wings may be an apomorphic version of this trait ( Figure 1A). A similar argument can be made for pleural wing (serial) homologs, with the proximal leg segments with branches (exites) as the most plesiomorphic, followed by pleural plates of hexapods as a more derived state, and wings as the most apomorphic version of this trait ( Figure 1B). The dual origin hypothesis proposes that the most apomorphic versions of these two traits actually overlap ( Figure 1). This hypothesis is attractive as it can potentially unify the two competing hypotheses; however, both tergal and pleural hypotheses are also valid at this point. Further investigation into wing serial homologs (outlined below) will help differentiate these hypotheses.

## Challenges of the evo-devo-based strategy in identifying wing serial homologs

Although the evo-devo-based approach is a promising method to provide new insights into the origin of insect wings, there are several weaknesses. (i) The first and foremost criticism to the evo-devo approach is its tendency to rely on a limited number of marker genes when identifying certain tissue lineages. Is *vg* expression sufficient to claim wing serial homology? How can we differentiate the *de novo vg*-dependent tissues from the true wing serial homologs? What if other wing marker genes, such as *nub* and *apterous* ( *ap*) ^47^, disagree with *vg* expression? (ii) Although several new wing serial homologs have been identified through evo-devo studies, it is far from being able to fully reconstruct a transition series for wing evolution. Below, we will discuss what we can do to go beyond simply identifying wing serial homologs and delve further into the developmental and genetic mechanisms that have facilitated the evolution of insect wings. ****

## Analyzing the wing gene network in wing serial homologs

Is *vg* expression sufficient to identify wing serial homologs? The answer is probably “no”. Although *vg* has been very useful in identifying wing serial homologs, it is risky to rely on just one marker because of the pleiotropic nature of the marker genes. Therefore, investigating *vg* expression will be useful as the first step to identify potential wing serial homologs, but it is also important to analyze the expression of additional wing marker genes. *nub* and *ap* are two popular wing marker genes; however, many more genes have been identified as being involved in wing development in *Drosophila*, some of which may be used as additional wing markers. Of particular interest are the genes involved in anterior/posterior wing patterning ^42^ and the genes involved in wing vein patterning ^48– 51^. To our knowledge, these genes have never been used to investigate the development of wing serial homologs or to identify possible wing homologs in non-insect arthropods. In addition, given the pleiotropic nature of the transcription factors and signaling molecules important for wing development, it is crucial to investigate not only the gene expression repertoire but also the gene regulatory network to assess wing serial homology and to understand the evolutionary relationships between the identified wing serial homologs. Recent advancement in genetic techniques in insects (such as CRISPR/Cas9 ^52, 53^) may allow us to investigate the regulatory hierarchy among wing genes in various non-model arthropods.

What if, then, the wing marker genes disagree with each other? Analyzing the gene regulatory network in a wide taxonomy will help identify which part of the network is ancestral and which part is lineage-specific. Also, we may need to consider which genes in the gene regulatory network provide more valuable information when evaluating homology. For example, *vg* may be more reliable than other marker genes, as *vg* appears to be the only gene whose expression can induce ectopic wing structures in *Drosophila* ^29, 30^. However, this “master gene” aspect of *vg* (and lack thereof in other genes) needs to be tested in other insects.

A tissue with the expression of *vg* and of other wing marker genes could have emerged via co-option. It is a challenge to differentiate these tissues from true wing serial homologs. As discussed above, we believe that Hox analysis will be powerful to exclude *de novo vg*-dependent tissues. Hox LOF mutations allow for transformations among serially homologous structures, while it is less likely that Hox LOF mutations can cause homeotic transformation between the original and the *de novo* structures evolved via co-option. For instance, the horn of *Onthophagus* beetles requires the leg gene network for its formation, but it does not transform into the leg upon Hox LOF mutation ^22, 23^. Thus, it is critical to assess the ability of a tissue to transform into the wing upon Hox mutation before determining whether the tissue is a wing serial homolog.

## Delving into the molecular and developmental mechanisms behind the evolution of insect wings

Identifying wing serial homologs has been quite helpful to gain new insights into the origin of insect wings; however, it is still largely elusive how insect wings have evolved. What can we do to go beyond identifying wing serial homologs and delve further into the molecular and developmental mechanisms that have facilitated the evolution of insect wings? One key direction is to study the development of wings and wing serial homologs in various species in detail. Previous expression analyses in *Tribolium* suggest that the primordia that give rise to wings and wing serial homologs are induced similarly in each segment during embryogenesis ^33, 54^. However, it is currently unclear how these possible primordial tissues contribute to the formation of wings and wing serial homologs and how these primordia differentiate into very different tissues over the course of development. By studying the molecular and developmental mechanisms that orchestrate the differentiation of wing serial homologs from wings (and vice versa), we will be able to identify the mechanisms that are operating uniquely in the winged segments.

Several approaches are useful for this direction. For example, tracing the development of wings and wing serial homologs will help us determine the developmental events that differentiate wings from other wing serial homologs and also will allow us to investigate the developmental origin of wings and wing serial homologs (such as tergal or pleural or both). Comparing gene regulatory networks between wings and wing serial homologs will also be useful to reveal the changes in gene regulation that have been crucial to the evolution of wings. Along the same line, transcriptome comparison between wings and wing serial homologs will allow us to comprehensively identify genes expressed differentially between these tissues. Another intriguing approach is to induce a “wing serial homologs-to-wing” transformation series. Analyzing these transformed tissues (their morphology, development, and gene expression) may help us reconstruct an evolutionary transition series from the origin tissue to the functional wing. Combining these approaches will be powerful to gain insights into the origin of insect wings. In fact, some of these approaches have been used in a recent study using *Oncopeltus*, which led the authors to identify a set of genes unique to the true wing when compared with the ectopic T1 wing created by Hox knockdown ^39^. This study has provided interesting insights into how ventral components have contributed to the evolution of insect wings ^39^.

**Figure 2.**
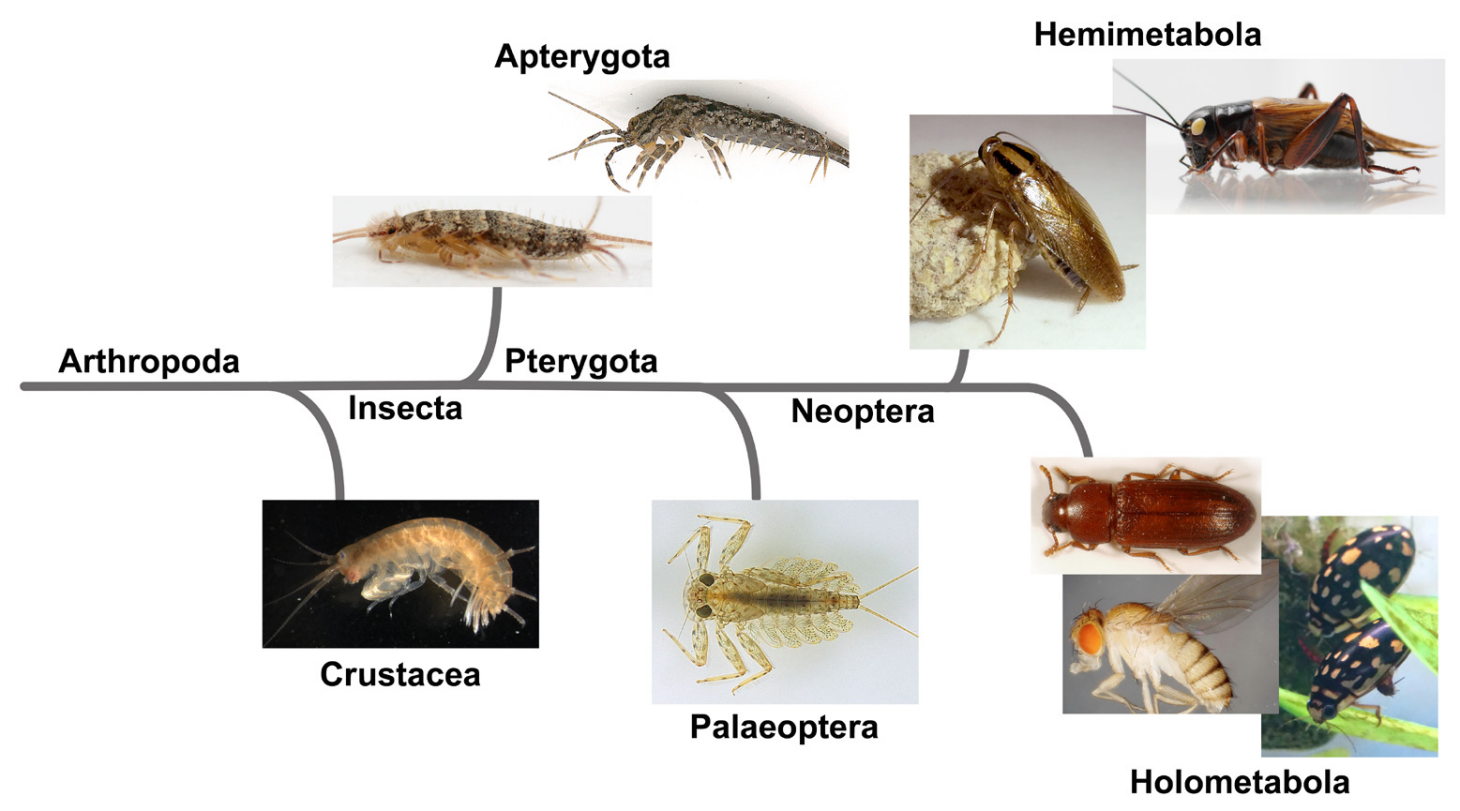
Various arthropod species that can be used for the evolutionary developmental study of insect wing origin. The species depicted here are as follows: Crustacea: the amphipod, *Parhyale hawaiensis*; Apterygota: the silverfish, *Thermobia domestica* and the bristletail, *Pedetontus unimaculatus*; Palaeoptera: the mayfly, *Epeorus latifolium*; Hemimetabola: the German cockroach, *Blattella germanica* and the cricket, *Gryllus bimaculatus*; Holometabola: the red flour beetle, *Tribolium castaneum*, the diving beetle *Thermonectus marmoratus*, and the fruit fly, *Drosophila melanogaster*.

The above approaches are somewhat technically demanding and thus likely need to be performed in relatively established model insects (such as *Tribolium*, *Blattella*, and *Oncopeltus*). However, some classic studies hint that it may be worth applying at least some of these approaches to non-model insects. For example, it has been reported that wing tissue arises at a lateral (pleural) position and migrates dorsally to merge into the tergolateral margin during the nymphal stages in a dragonfly, which may support the pleural (or perhaps dual?) origin of insect wings ^2, 55^. Similar dorsal migration of wing primordia from the lateral region has been described in the embryos of some hemimetabolous insects, such as the cockroach, *Periplaneta americana* ^2, 56^. Some of these situations may partially recapitulate phylogeny; thus, studying the development of wings and wing serial homologs in these species will be helpful to gain more information in regard to the evolutionary transitional state(s) from the origin tissue to the wing. However, caution must be taken when making this argument, as a developmental process unique to a certain lineage (apomorphic) can superficially mimic possible plesiomorphic conditions. For example, in the past, the wing disc formation from the leg disc in *Drosophila* was used to support a leg origin of insect wings ^2^. However, this invaginated imaginal disc formation is a highly derived trait even in holometabola. In addition, the so-called “wing” and “leg” discs produce not only the appendages but also the entire adult epidermis (the wing disc produces the dorsal half, and the leg disc produces the ventral half ^43^). Therefore, the *Drosophila* situation likely does not reflect an ancestral state. In summary, detailed studies on the development of wings and wing serial homologs in various species will help us identify key developmental and genetic events that have facilitated the evolution of insect wings ( Figure 2).

## Moving beyond the winged insects

As mentioned, previous expression analyses for wing marker genes in non-insect arthropods ^44, 45^ and in non-winged hexapods ^46^ have shown that evo-devo analyses can provide critical information for the study of insect wing origin, thus establishing a basis for the expansion of our analysis of insect wing origin beyond the winged insects and even beyond Insecta. An essential next step in this direction is to analyze the function of wing gene homologs and their genetic interaction in a diverse array of arthropod taxa ( Figure 2). For example, the tergum and stylus (a pleural structure) of a non-winged insect (bristletail) have been found to express *vg* ^46^. It will be interesting to investigate how much of the gene regulatory network operating in these tissues is shared with insect wings. Functional analyses in various crustaceans will also be beneficial to further identify tissues that share ancestry with the insect wing (that is, wing homologs). Several leg branches (homologous to a pleural lineage) in the brine shrimp and the crayfish have been shown to express some wing marker genes ^44^, but their functional dependency on wing marker genes, including *vg*, still needs to be tested. In addition, it is yet to be determined whether other tissues in these crustaceans (such as terga) also share gene regulatory networks with the insect wing. Furthermore, given the vast diversity and the possible polyphyletic nature of the crustacean order ^57, 58^, it will be critical to analyze more crustacean species. Myriapoda (millipedes and centipedes) is another taxon that may provide interesting insights in regard to identifying tissues homologous to insect wings. The possible wing homologs in Myriapoda have not yet been investigated. However, some myriapods possess elaborated tergal expansions reminiscent of paranotal lobes. Therefore, it would be interesting to investigate whether these structures (and other tissues such as parts of the leg) have dependency on genes homologous to insect wing genes. The myriapod lineages are even more basal on the arthropod phylogeny than crustaceans ^58^, and thus identification of potential wing homologs in the myriapod lineages can provide crucial information as to which tissues have given rise to insect wings. In summary, functional analyses for genes homologous to wing genes in a diverse array of arthropod taxa will lead us to a better understanding of what tissues are homologous to wings in these lineages, which will help us further evaluate the wing origin hypotheses from an evo-devo perspective.

## Note added in revision

While we were revising this manuscript, Prokop *et al.* reported very intriguing findings that provide support for a dual origin of insect wings from the paleoentomological point of view ^59^. Collaboration among various fields, including paleontology and evo-devo, will be fruitful to tackle this century-old question regarding the evolutionary origin of insect wings.

**Box 1. Glossary**

**Dorsoventral insect anatomy:** The insect thoracic and abdominal body wall can be subdivided into three distinct regions: dorsal (tergum), lateral (pleuron), and ventral (sternum).

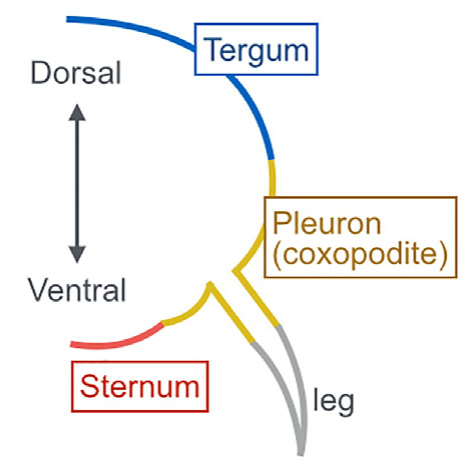

**Tergum (pl. terga):** A large plate that forms the dorsal body wall in the thoracic and abdominal segments. The thoracic tergum is called **notum**.

**Pleuron (pl. pleura):** The lateral portion of insect thoracic and abdominal segments that consists of several pleural plates. The pleural plates are thought to have stemmed from ancestral proximal leg segments that have fused into the body wall of extant insects.

**Sternum (pl. sterna):** The large ventral plate of insect thoracic and abdominal segments.

**Tergal origin hypothesis (also known as paranotal lobe hypothesis):** This hypothesis proposes that wings originated from expansions of the dorsal body wall (terga).

**Pleural origin hypothesis (also known as gill or exite hypothesis):** This hypothesis proposes that wings evolved from pleural (lateral body wall) tissues and their associated branches (exites).

**Dual origin hypothesis:** This hypothesis proposes that both tergal and pleural components have contributed to the evolution of insect wings.

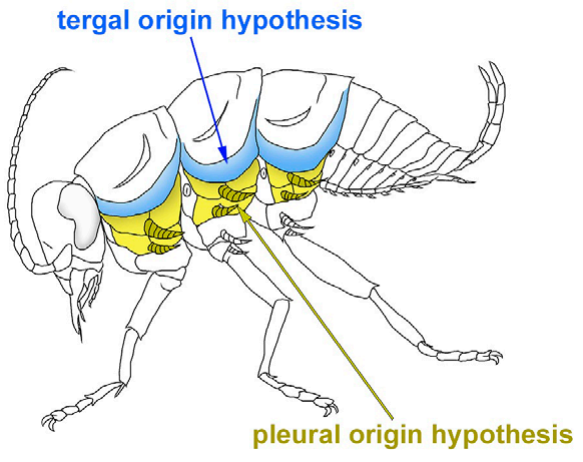

**Carinated margin:** A lateral expansion of dorsal body wall (tergum) in the first thoracic segment in beetles. This structure and several pleural plates have been identified as wing serial homologs ^33^.

**Gin-trap:** A defensive structure formed at the lateral portion of the abdominal segments in some coleopteran pupae. This structure has been identified as a wing serial homolog ^32^. The gin-trap structure is also observed in lepidopteran pupae; however, the evolutionary and developmental relationship between coleopteran and lepidopteran gin-traps is currently unknown.

**Helmet:** An often highly decorated structure of the first thoracic segments in treehoppers. This structure has been proposed as a modified wing of the T1 segment in treehoppers ^35^, which was later disputed from a morphological standpoint ^37, 38^. Clark-Hachtel and Tomoyasu have proposed that the treehopper helmet is serially homologous to the dorsal (tergal) wing serial homologs but not lateral (pleural) wing serial homologs; therefore, the helmet is “partially” serially homologous to wings ^3, 33^.

**Apomorphy:** Within a group of organisms that have common ancestry, an apomorphy is a trait that is diverged or novel compared with the ancestral state.

**Plesiomorphy:** Within a group of organisms that have common ancestry, a plesiomorphy is a trait that is maintained/conserved throughout the course of evolution from a common ancestor.
